# Supplementary material for: Patterns of Intron Gain and Loss in Fungi
Source: PLoS Biol. 2004 Nov 30;2(12):e422. doi: 10.1371/journal.pbio.0020422 (PMC532390; doi:10.1371/journal.pbio.0020422)
Supplement: Table S1 — Also available at http://genes.mit.edu/NielsenEtAl/. (4.3 MB ZIP). [file pbio.0020422.st001.zip › NielsenEtAl/html/14.html]

AN1779.1.NCU05972.1.MG00597.1.FG09310.1


```
 CLUSTAL W (1.82) Multiple Sequence Alignments - Introns Inserted


Sequence 1: NCU05972.1	706 aa
Sequence 2: MG00597.1	651 aa
Sequence 3: FG09310.1	629 aa
Sequence 4: AN1779.1	606 aa
Alignment Length: 728 aa
Number Identitical Residues: 130 aa
Alignment Score (without introns) 7551


MG00597.1 	MSSRS-------RVPLSSNPNAANSPMR---PMAVAHP---KPVKRSHASNQREELYGQP
NCU05972.1	MSSTTTTAAGTKRAPLSDNPNAANSPLRGSSATALALQGSKKAAVRSHADQLREEPYGQP
FG09310.1 	MSSRR--------GPLTNNPNVANSPLRG--VSALAGY---AKQKRSYATVQREETYGQP
AN1779.1  	MSTRR---------PLANVPNATNSPHRVGLVPAKRPR---------TTSAQIDIPYGQP
          	**:           **:. **.:*** * .   *              :    :  ****

MG00597.1 	PPAKKQMISN--------PSTYR-----VTARSPVKAR------------------TTTH
NCU05972.1	PPAKRQMIEHGGQRAAASPTSSRSNVSNITRKASTISRPIDRERPERASSRSGTTVATST
FG09310.1 	PPLKKQVLDNG-----------------VQRPVRSPTR------------------STRT
AN1779.1  	PPKKQVVDGAGAENQPMSQTKFAALQGTDPKLFTRKTN-------------------NAQ
          	** *: :   ...  . : :.  :  ..        :.                   .  

MG00597.1 	TITQSRAVGGHRDRDAASQQHVKAQLADEDVNDFKKWAAAQKQQFVKHVFYLDNLPQEVL
NCU05972.1	TTRAERLYAASTTTTTTSTTTTTDRITAKELEDLSIWQTSMRSRFHKMVFYFESIPDEQR
FG09310.1 	QVLVQRGTTRPSTKERSSRTAPTN-VARDMDNEKEAWKKHHRAKFPKMVFYFESIPDDVR
AN1779.1  	PSAFEKKLVAARDKERQPQKATKQEKPPAENISIRQWQRHYRKAFPHFVFYFDAVPIDVR
          	    .:           .    .   .     .   *    :  * : ***:: :* :  

MG00597.1 	PKIERQIAALGA0REDKFFSISISHVVTTRPIPQPQT--SQKLKLDVSTKNDREDQEPQT
NCU05972.1	HKLAKQVAQLGA0REEKFFSIEITHVVTTRPIPPVKP--VQDENQPVTNTQSTSEQ-PKT
FG09310.1 	ARLTKKVTYLGA0QQEPFFSISVTHVVTTRSIPPERPEGHQEQEHNHRDDHHESEEQPQT
AN1779.1  	SKCSRQVIALGA0REEKFFSRLVTHVVTSRPIPPEIDRRAQTGHTQDTPNESAGDG----
          	 :  :::  *** ::: ***  ::****:*.**       *  .      .   :     

MG00597.1 	INPSLLSRNLDSNCAKRKLFDTHPAGRRPPIQLGEDVVRRPKAPRQQNDILGRALTMGKK
NCU05972.1	IDPSLLNRNGDS--VRRKLIFDKPSLRKSSVQPQEETMPKPKAR--STDILHRARDMGKK
FG09310.1 	INPSLLNRNTEA---RRKLLSEFRNAPARTQQIDDPTKRTRGTR--NNDVLHKAREMGKK
AN1779.1  	------------------AMLQTVNPAELEMHLHLAVCPKREQS---QDVLHRAREMGMK
          	                   :           :    .           *:* :*  ** *

MG00597.1 	IWSLEKMQRILDMLTESDPYRAATLGQGGR-----TAKTVEQ-SNPLQMLNKERVSGPSD
NCU05972.1	IWSLDKLQRILGMVLETDLYKAALLGRGHTPAAADRAKARQQTANVLQMLQNERVHGPSD
FG09310.1 	IWSLEKFQNMLSVLLEVETSHASYSSRSASTR---SHYGNPREPNLLQLLHNERVNGPSD
AN1779.1  	IWAVEKLQRMIATINDIDLTNGSGHSTRNNAAG---SQTKSRGKDDLSQVLQNELNGPSD
          	**:::*:*.::  : : :  ..:  .    . .        :  : *. : ::.: ****

MG00597.1 	RDPTASTKEMHLFKGPFIYIYDIEQKQRPIMAREYAKVADKMKGDWPQFRASGSGRCPFV
NCU05972.1	RDPTVVTKDIHYFKGPYIYVYDIEEKTRPIMVREYHRVADKKDGEWPQFRVASQGRCPFV
FG09310.1 	RDPTAVHRELTYFKGPHIYVWDMDEKNKPIMVREYPKVTNKSDGEWPQFRSVGNGRCPFV
AN1779.1  	RSHLSVLKDLVPFKGPFIYVHDMDEKTRPVMVREYPKVARRQDGVWPQFRSAPLGKCPFI
          	*.     :::  ****.**: *:::* :*:*.*** :*: : .* *****    *:***:

MG00597.1 	EDHDPEKHSKKER-----EVARQAAVNAAVGSAPKLQPPTQIARPKPITGKRPLAEMSEV
NCU05972.1	EDYDPPERTRREKTQTKEKVVKAADAAAATEKATKTLQPPEVPSPKPVTGKRTLKEMEAP
FG09310.1 	EDHDVPDKDHRRQ------REQEKARQAKREEAAHAIKPPQVSMPKPVTGKRTIAEMEE-
AN1779.1  	EDVPSKREIERQR-------ARQEKEKKEFIKPAVPQSKHTAVEPRNEENLPLKKETSPA
          	**     . .:.:        :         ...          *:   .     * .  

MG00597.1 	STNRILFPSKTTTGGNALMDPTQGSKLSKEDALSNAFISTNRSRPARIFAG-EPVASGLQ
NCU05972.1	NDRATEVVDDAPETVESAKEPTPSVDFRSQ----NAFMS-HTTKVGRLVAG-EPVASGIQ
FG09310.1 	GQNRIRGATPTDVFNPVKVAMSKQVDVRPQ----NAFTS--RAAAGRLFAG-EPVASGVQ
AN1779.1  	EGDELAPHCTRQETPDAPQGLPLSPKKSSE-----SFIPPQLNRKGPFYHGREPAASGVQ
          	                     .   .   :     :* ..     . :  * **.***:*

MG00597.1 	ASNITSAIRSQMISSTTGTIASNKSGVSKEVLGLQRKVLAKSG---SISQDPSSRRHAEM
NCU05972.1	ANKLTSAIRSQMVSSTTGVLG-AKAGISKEVLGLQRKVLQKAGG-PSLSQDSGVRRLAEI
FG09310.1 	PSNITSAIRSQMISSTSGVNG-AKAGTSKEVHGLQRKVLQKAA---PASFDVSSRRLAEV
AN1779.1  	PSNITSAIRSQMVSSTAAAPG-AKAGLSKEVHELKRKVLEKSHVGYSTNVAQPYRALDTA
          	..::********:***:.. .  *:* ****  *:**** *:  . . .     *     

MG00597.1 	SVDSGPLSRSTSLGNPR-----LIFEGEETQH----------RRTHSVPSKLQPKPKKRD
NCU05972.1	GVDAAQCTRAASLGNPAKPPMPGIWEDDEARKQEQAKKERKLRRTASTP-VPQTKPKPRD
FG09310.1 	SMDVAS-SRSTTMGRHTSR--PAEVQEEEAQKT--------ERRTQSQP--LKSK---KD
AN1779.1  	TAERTEKTNSCKSSRPDKLG---NIEEETTQSEATDS--TKSRTILRKSGEQRKKERRRD
          	  :    :.: . ..  .       : : ::     .   . *     ..  : *   :*

MG00597.1 	PKPGYCENCQDKFADFEE0HIQSRTHRKFADNDENWEDLDRLLQQLERQP-LPHHHLYSD
NCU05972.1	PKPGYCENCQDKFEDFDA0HIVSRKHRRFAENDDNWAQLDALLGKLSRAPREPRYRSVGD
FG09310.1 	LKPGYCENCQDKFRDFDE0HILSRKHRKFAENDDNWIELDSLLSQIKRMP---KYAPGSD
AN1779.1  	PKPGYCENCRDKFDDFDE0LERP------LKDEYDYV-----------------------
          	 ********:*** **:     .       .:: ::                        

MG00597.1 	ETVEQNYDQY
NCU05972.1	ERWQ------
FG09310.1 	EEEAW-----
AN1779.1  	----------
          	
```
